# A comparison of the effects of resistant starch types on glycemic response in individuals with type 2 diabetes or prediabetes: A systematic review and meta-analysis

**DOI:** 10.3389/fnut.2023.1118229

**Published:** 2023-03-27

**Authors:** Jennifer E. Pugh, Mingzhu Cai, Nunzia Altieri, Gary Frost

**Affiliations:** Section for Nutrition Research, Department of Metabolism, Digestion and Reproduction, Faculty of Medicine, Imperial College London, Hammersmith Campus, London, United Kingdom

**Keywords:** resistant starch, type 2 diabetes, glucose, insulin, food structure

## Abstract

**Background:**

Type 2 diabetes (T2D) diagnoses are predicted to reach 643 million by 2030, increasing incidences of cardiovascular disease and other comorbidities. Rapidly digestible starch elevates postprandial glycemia and impinges glycemic homeostasis, elevating the risk of developing T2D. Starch can escape digestion by endogenous enzymes in the small intestine when protected by intact plant cell walls (resistant starch type 1), when there is a high concentration of amylose (resistant starch type 2) and when the molecule undergoes retrogradation (resistant starch type 3) or chemical modification (resistant starch type 4). Dietary interventions using resistant starch may improve glucose metabolism and insulin sensitivity. However, few studies have explored the differential effects of resistant starch type. This systematic review and meta-analysis aims to compare the effects of the resistant starch from intact plant cell structures (resistant starch type 1) and resistant starch from modified starch molecules (resistant starch types 2–5) on fasting and postprandial glycemia in subjects with T2D and prediabetes.

**Methods:**

Databases (PubMed, SCOPUS, Ovid MEDLINE, Cochrane, and Web of Science) were systematically searched for randomized controlled trials. Standard mean difference (SMD) with 95% confidence intervals (CI) were determined using random-effects models. Sub-group analyses were conducted between subjects with T2D versus prediabetes and types of resistant starch.

**Results:**

The search identified 36 randomized controlled trials (*n* = 982), 31 of which could be included in the meta-analysis. Resistant starch type 1 and type 2 lowered acute postprandial blood glucose [SMD (95% CI) = -0.54 (–1.0, –0.07)] and [–0.96 (–1.61, –0.31)]. Resistant starch type 2 improved acute postprandial insulin response [–0.71 (–1.31, –0.11)]. In chronic studies, resistant starch type 1 and 2 lowered postprandial glucose [–0.38 (–0.73, –0.02), –0.29 (–0.53, –0.04), respectively] and resistant starch type 2 intake improved fasting glucose [–0.39 (–0.66, –0.13)] and insulin [–0.40 (–0.60, –0.21)].

**Conclusion:**

Resistant starch types 1 and 2 may influence glucose homeostasis *via* discrete mechanisms, as they appear to influence glycemia differently. Further research into resistant starch types 3, 4, and 5 is required to elucidate their effect on glucose metabolism. The addition of resistant starch as a dietary intervention for those with T2D or prediabetes may prevent further deterioration of glycemic control.

## Introduction

Type 2 diabetes (T2D) is the ninth leading global cause of death and diagnoses are predicted to reach 643 million by 2030 (1, 2). T2D is defined as chronic elevations in blood glucose concentrations due to insulin resistance or impaired insulin secretion (3). Diet and lifestyle interventions aim to alleviate symptoms and complications of T2D by preventing substantial elevations in postprandial blood glucose (4). Averting prolonged periods of hyperglycemia may lower the risk of oxidative tissue damage and the subsequent development of comorbidities, such as cardiovascular disease (5).

Resistant starch cannot be digested by endogenous amylases in the small intestine and reaches the distal gut where it can be fermented by the colonic microbiota (6). Resistant starch is categorized into five types (7). Naturally occurring resistant starch is found enclosed within plant cells (resistant starch type 1) or in high-amylose species of grains (resistant starch type 2) (7). Alterations to the starch molecule may increase resistant starch content; cooking and cooling starchy food forms retrograded resistant starch type 3, chemical modification of starch produces resistant starch type 4 and starch-lipid complexation forms resistant starch type 5 (8–10).

The industrial or domestic processing of food structures to reduce particle size may increase plant cell wall permeability, lowering resistant starch type 1 content (11, 12). Reducing processing to protect plant cell integrity preserves the cell wall components which inhibit α-amylases and provide a physical barrier to protect intracellular starch from digestion (13). Plant cell integrity lowers starch available for hydrolysis in the small intestine, effectively dampening postprandial glycemic response.

Resistant starch can dilute the digestible starch content of a meal, lowering the glycemic load and attenuating postprandial glucose and insulin response. Resistant starch remains unabsorbed in the upper gastrointestinal tract and is delivered to the colon where it can favorably alter gut microbial composition. Resistant starch has been associated with increased *Roseburia*, *Faecalibacterium*, *Akkermansia*, and *Bifidobacteria*; bacterial populations which have been negatively correlated with T2D (14, 15). However, this may be dependent upon the type and plant source of the resistant starch (16). Fermentation of resistant starch by colonic bacteria results in the production of short-chain fatty acids (SCFAs) and secondary bile acids (17, 18). SCFAs, specifically butyrate, and some secondary bile acids are positively associated with insulin sensitivity (19–23). Furthermore, SCFAs promote the production and secretion of glucagon-like peptide 1 (GLP-1), an incretin which modulates glucose-stimulated insulin release (24, 25). This evidence suggests that the delivery of fermentable resistant starch to the colon plays a role in prolonged improvements to glucose homeostasis and insulin sensitivity (26, 27).

Interventions with intact plant cell structures, preserving resistant starch type 1, significantly dampened postprandial glucose and insulin responses in healthy individuals (28). Acute studies in healthy volunteers have demonstrated attenuated postprandial glucose and insulin excursion after interventions supplementing resistant starch type 2–4 or substituting it for digestible starch (29–32). Furthermore, a recent meta-analysis concluded that resistant starch significantly lowered fasting blood glucose and insulin resistance in healthy subjects (33). However, few studies with interventions of this type are conducted on interventions of this type in individuals with impaired glucose control.

Foods with resistant starch type 1 may differentially affect glycemia compared to resistant starch 2–5. Although, cell wall permeability may be altered by thermal or physical processing (i.e., cooking, milling, or mastication), which may alter the availability of starch in the distal gut. However, current literature appears to support that when the structural integrity of the plant cell is maintained, an improved glycemic response is seen (12, 34). Furthermore, resistant starch type 1 may inhibit starch digestion *via* multiple mechanisms, these have been comprehensively summarized by Xiong et al. (35).

There is an urgent need for cost-effective and accessible methods of maintaining euglycemia to reduce the risk of complications for individuals with T2D and prevent further glycemic deterioration in those with prediabetes. This review and meta-analysis aims to compare the effects of the type of resistant starch (type 1 versus 2–5) on glycemia in individuals with impaired glucose regulation and to identify any differential effects between study populations (i.e., T2D versus prediabetes).

## Methods

This systematic review was conducted following the Preferred Reporting Items for Systematic Reviews and Meta-analysis (PRISMA) guidelines (28). The protocol for this systematic review was accepted to the prospective register of systematic reviews (PROSPERO) on February 1st, 2021, with the reference number CRD42021233918.

### Eligibility criteria

The population, intervention, comparator, outcome, and study design (PICOS) criteria were used to guide the search strategy and study selection (Table 1). In this review and meta-analysis, the population of interest were adults (18–65 years), with type 2 diabetes or prediabetes (including those with metabolic syndrome), and without other chronic conditions. The interventions were resistant starch types 1–5 (both naturally occurring and in the form of a food supplement) and starchy foods that were unprocessed or had large particle sizes. This intervention was compared to relevant and ideally nutritionally-matched starchy foods. The outcomes explored were postprandial glucose and insulin incremental area under the curve (iAUC), fasting glucose and insulin, measures of insulin sensitivity: homeostatic model assessment of insulin resistance, beta-cell function or insulin sensitivity (HOMA-IR,%B, or %S), glycated hemoglobin (HbA1c), GLP-1 and gastric inhibitory peptide (GIP) concentrations. Studies of any duration were included, with a duration of <1 day considered an acute study and a duration of >1 day, a chronic study. Only randomized controlled trials were included.

**TABLE 1:** Population, intervention, comparator, outcome, and study design (PICOS) criteria for study eligibility.

	Inclusion	Exclusion
Participants	Adults, >18 years old, diagnosed with type 2 diabetes or prediabetes (as diagnosed by any criteria)	Patients with prediabetes/T2D AND other chronic conditions (nephropathy, liver failure, coronary heart disease)
Intervention	Resistant starch type 1–5 (naturally occurring and purified), starch-containing foods with structural properties which reduce starch digestibility (e.g., high amylose, large particle size)	High fiber interventions, irrelevant interventions
Comparator	Starch/other carbohydrates (non-resistant), food structures which increase starch digestibility (e.g., smaller particle size)	Control conditions with non-comparable nutrient composition
Outcome	Markers of glycemia: fasting glucose and insulin, glucose and insulin iAUC, measures of insulin sensitivity (HOMA-IR, HOMA-B/S%), HbA1c%, GLP-1, and GIP	Studies which did not measure outcomes of interest
Study design	Randomized controlled trials	Reviews, conference abstracts, dissertation abstracts, lectures, information pieces, study registers, and *corrigendum*

GLP-1, glucagon-like peptide-1; GIP, gastric inhibitory peptide; HbA1c, glycated hemoglobin; HOMA-%B, homeostatic measurement of beta cell function; HOMA-%S, homeostatic measurement of insulin sensitivity; iAUC, incremental area under the curve; T2D, type 2 diabetes.

### Search strategy

The literature search was conducted across PubMed, SCOPUS, Ovid MEDLINE, Cochrane, and Web of Science databases for any study-related documents published before 5th May 2022. Specific search terms are found in Supplementary Table 1.

### Screening and data extraction

The authors independently reviewed all article titles and abstracts using Covidence systematic review software (Veritas Health Innovation, Melbourne, VIC, Australia). The researchers of registered clinical trials were contacted to provide access to the raw data when trials were deemed relevant. Potentially included studies underwent full-text screening by the three reviewers (JEP, MC, NA), independently using the inclusion and exclusion criteria found in Table 1. Any conflicts during screening or full-text review were discussed until a consensus was reached. After screening, the PICOS data from each study were extracted by the reviewers. Data found within figures were extracted using WebPlotDigitizer^1^.

### Risk of bias assessment

The risk of bias (ROB) of eligible studies has been independently assessed by three reviewers (JEP, MC, NA) using the Revised Cochrane ROB tool. This tool identifies the level of risk of bias during the randomization process, deviations from the intended intervention, missing outcome data, measurement of outcomes, and selection of the reported result. The studies are categorized to have a low, some concerns or high risk of bias. Randomized controlled crossover studies were assessed for ROB arising from period and carryover effects of crossover studies (domain S). In instances where included studies were not crossover trials, this section was left blank.

### Data analysis

Studies were categorized by study duration and resistant starch type. Data were recorded and organized in an Excel spreadsheet. Mean and standard deviations were calculated if they were not available in the reports. Standard errors of means were converted to standard deviations. If there were multiple intervention groups, means and SDs were pooled according to the Cochrane method (29). Review Manager Version 5.4 (the Cochrane Collaboration, Software Update. Oxford, UK) was used for meta-analyses using the random effects model, under generic inverse variance. Pooled standard mean differences (SMDs) with 95% confidence intervals (CIs) were calculated for outcomes of interest: fasting glucose, fasting insulin, postprandial glucose, and insulin, HOMA-IR, HOMA-%S, HOMA-%B, HbA1c, GLP-1, and GIP. Random effects models and SMD were used due to differences in the type, duration and design of the included studies. Furthermore, SMD is more generalizable and less heterogeneous than MD (30). Statistical significance was determined by a *p*-value of <0.05. Heterogeneity was quantified with the I^2^ statistic and *p*-values of <0.05 were statistically significant. Where possible, subgroup analysis was used to explore differences in effect between different types of resistant starch and between subjects with T2D versus prediabetes. Sensitivity analyses were conducted by omitting studies with a high risk of bias. Publication bias was assessed using funnel plots and the regression test for Funnel plot asymmetry (“Egger’s test”), in JASP (Eric-Jan Wagenmakers, Amsterdam, Netherlands).

## Results

**FIGURE 1:**
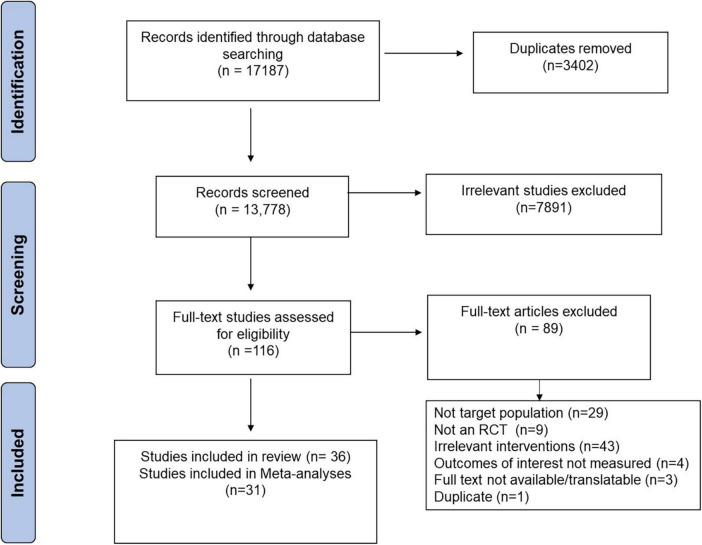
Preferred Reporting Items for Systematic Reviews and Meta-analysis (PRISMA) flow diagram of the literature search and screening process.

A summary of the literature search and screening process is found in Figure 1. The search identified 17,187 publications. Specifically, 6,871 from SCOPUS, 5,783 from OVID, 4,060 from Web of Science, 378 from PUBMED, and 95 from Cochrane. A total of 116 publications were identified for full-text review. Studies excluded after the full-text review (*n* = 89) and the reasons for their exclusion are found in Supplementary Table 2. No response was received from the researchers that were contacted for access to data from registered RCTs. This review includes 36 papers (*n* = 982), 31 of which could be incorporated into the meta-analysis. The five studies were excluded because they did not report standard error or standard deviation (36), or reported mean difference from baseline for measurements of interest (37–40). For studies with multiple intervention groups (41–44), the means and SDs of the interventions were pooled (29). No studies were identified that investigated resistant starch type 5. A summary of characteristics for included studies is found in Supplementary Table 3. Sensitivity analyses where initial conclusions were altered and funnel plots that indicate publication bias are found in the Supplementary material.

### Risk of bias in included studies

**FIGURE 2:**
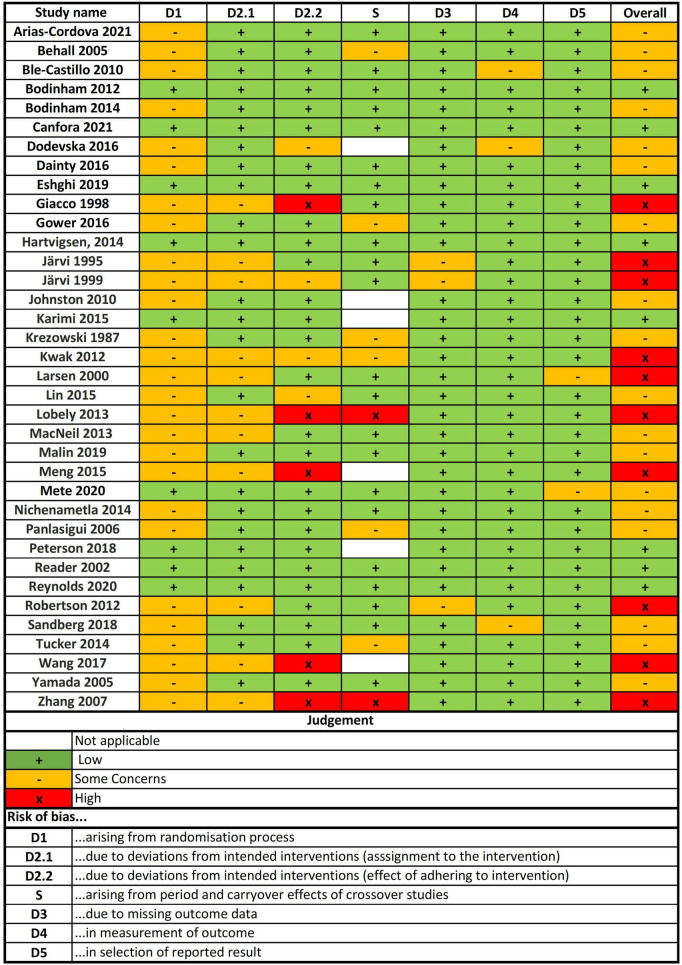
Risk of bias of included studies.

After the risk of bias analysis, 28% of included studies had a high risk of bias, 50% had some concerns and 22% had a low risk of bias (Figure 2). The main concerns were risk of bias arising from the randomization process (D1), and risk of bias due to deviations from the intended intervention (assignment to the intervention and effect of adhering to the intervention) (D2).

### Acute resistant starch and glycemia

**FIGURE 3:**
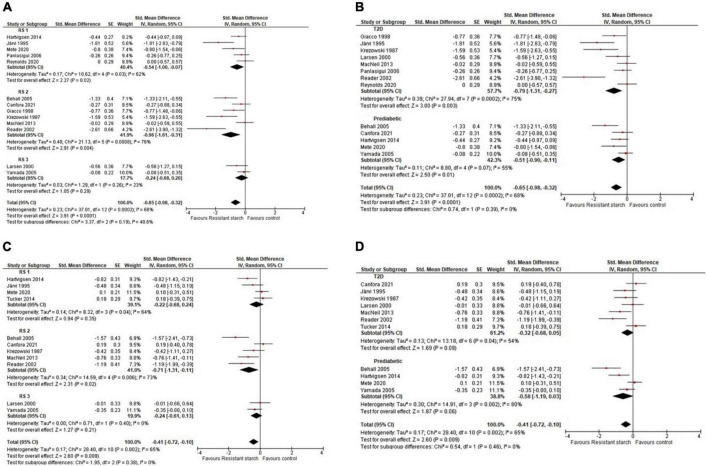
Std. Mean Difference: acute resistant starch types 1–4 intake on postprandial glucose incremental area under the curve (iAUC), with subgroup analysis by resistant starch type **(A)**, postprandial glucose, with subgroup analysis for prediabetes and type 2 diabetes (T2D) groups **(B)**. Postprandial insulin response iAUC, with subgroup analysis by resistant starch type **(C)**, postprandial insulin, with subgroup analysis for subgroup analysis for prediabetes and T2D **(D)**.

In a meta-analysis of 14 studies (*n* = 177) (41–43, 45–55), resistant starch significantly lowered postprandial glucose response [SMD (95% CI) = –0.65 (–0.98, –0.32)]. Subgroup analyses determined that resistant starch type 1 and 2 lowered postprandial glucose response [–0.54 (–1.0, –0.07)] and [–0.96 (–1.61, –0.31)], whereas resistant starch type 3 had no significant effect [–0.24 (–0.68, 0.20)] (Figure 3A). In subgroup analyses, resistant starch lowered postprandial glucose in participants with T2D [–0.79 (–1.31, –0.27)] and prediabetes [–0.51 (–0.9, –0.11)] (Figure 3B). In a meta-analysis of 11 studies (*n* = 142) (41, 43, 45–47, 49–51, 53–55), resistant starch significantly lowered postprandial insulin [–0.41 (–0.72, –0.1)], however, subgroup analysis determined that only resistant starch type 2 had a significant effect on postprandial insulin [–0.71 (–1.31, –0.11)], not resistant starch type 1 [–0.22 (–0.68,0.24)], or 3 [–0.24 (–0.61, 0.13)] (Figure 3C). In subgroup analyses, resistant starch did not affect postprandial insulin in subjects with T2D [–0.32 (–0.68, 0.05)] or prediabetes [–0.58 (–1.19, 0.03)] (Figure 3D). Interstudy heterogeneity was significant for both meta-analyses, postprandial glucose (I^2^ = 68%; *P* = 0.001) and postprandial insulin (I^2^ = 65%; *p* = 0.009). After conducting sensitivity analyses, acute resistant starch type 1 intake no longer had a significant effect on postprandial insulin response (Supplementary Figure 1). Publication bias was detected in the resistant starch type 1 and 2 in the postprandial glucose meta-analysis (*p* ≤ 0.05) (Supplementary Figures 2, 3).

### Chronic resistant starch and glycemia

**FIGURE 4:**
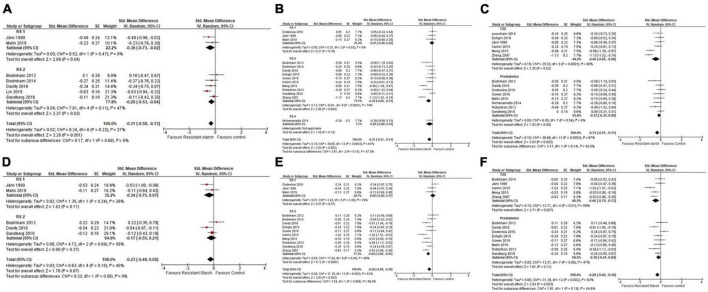
Std. Mean Difference: chronic resistant starch types 1–4 intake on postprandial glucose incremental area under the curve (iAUC), with subgroup analysis by resistant starch type **(A)**, fasting blood glucose with subgroup analysis by resistant starch type **(B)**, fasting blood glucose with subgroup analysis for prediabetes and type 2 diabetes (T2D) **(C)**, postprandial insulin iAUC, with subgroup analysis by resistant starch type **(D)**, fasting insulin with subgroup analysis by resistant starch type **(E)**, fasting insulin with subgroup analysis for prediabetes and T2D groups **(F)**.

In a meta-analysis of seven studies (*n* = 168) (56–62), resistant starch lowered postprandial glucose [–0.31 (–0.50, –0.13)]. Subgroup analysis indicated resistant starch type 1 [–0.38 (–0.73, –0.02)] and 2 [–0.29 (–0.53, –0.04)] had a significant effect on postprandial glucose (Figure 4A). In a meta-analysis of 14 studies (*n* = 410) (44, 56–61, 63–69), chronic resistant starch intake affected fasting glucose [–0.31 (–0.51, –0.11)]. Subgroup analyses indicate resistant starch type 2 affected fasting glucose [–0.39 (–0.66, –0.13)], whereas resistant starch type 1 [–0.03 (–0.29, 0.22)] and 4 [–0.29 (–0.68, 0.10)] did not (Figure 4B). In subgroup analyses, resistant starch lowered fasting glucose in participants with T2D [–0.46 (–0.85, –0.08)] but not prediabetes [–0.15 (–0.30, 0.00)] (Figure 4C). In a meta-analysis of five studies (*n* = 112) (56, 57, 59–61), resistant starch did not affect postprandial insulin response [–0.23 (–0.49, 0.02)] (Figure 4D). In a meta-analysis of 13 studies (*n* = 383) (44, 56–61, 63–66, 68, 69), resistant starch lowered fasting insulin concentrations [–0.29 (–0.49, –0.10)]. Subgroup analysis indicated that resistant starch type 2 had a significant effect [–0.40 (–0.60, –0.21)] but resistant starch type 1 did not [0.08 (–0.20, 0.37])] (Figure 4E). In subgroup analyses, resistant starch intake lowered fasting insulin in subjects with T2D [–0.46 (–0.79, –0.13)], not in subjects with prediabetes [–0.18 (–0.41, 0.04)] (Figure 4F). Sensitivity analyses did not alter initial conclusions. No indication of publication bias was seen in funnel plots. Heterogeneity was high for fasting glucose (I^2^ = 67%; *p* = 0.002) and fasting insulin (I^2^ = 62%; *p* = 0.03), but non-significant for other meta-analyses.

Of the studies which could not be included in the meta-analysis, three reported that the addition of resistant starch to the diet did not affect fasting glucose or insulin (36, 39, 40). One study (38), reported that resistant starch type 2 lowered fasting glucose but did not affect fasting insulin. Conversely, Ble-Castillo et al. (37), reported that resistant starch type 2 significantly reduced fasting insulin but did not affect fasting glucose.

### Resistant starch and incretin hormones

In a meta-analysis of five studies (*n* = 95) (43, 45, 57, 58, 61), resistant starch did not affect GLP-1 concentrations [0.01 (–0.24, 0.27) (Supplementary Figure 4A). In a meta-analysis of three studies (*n* = 40) (43, 45, 57) resistant starch did not affect GIP concentrations [–0.64, (–1.57, 0.30)] (Supplementary Figure 4B). Sensitivity analyses did not alter initial conclusions. Publication bias was detected for GIP (*p* ≤ 0.001) (Supplementary Figure 5).

### Resistant starch and markers of insulin sensitivity

Meta-analyses indicated that resistant starch did not affect HOMA-IR, (*n* = 222), (60, 61, 63–65, 68, 70), [–0.25 (–0.52, 0.003)] (Supplementary Figure 6A), HOMA-%B, (*n* = 66), (58, 60, 68, 71), [–0.36 (–1.08, 0.36)], (Supplementary Figure 6B), HOMA-%S (*n* = 61), (58, 60, 71), [0.32, (–0.06, 0.69)] (Supplementary Figure 6C) or HbA1c (*n* = 101), (56, 58, 64, 67) [–0.18, (–0.54, 0.17)] (Supplementary Figure 6D). Sensitivity analyses did not alter initial conclusions. Publication bias was detected for HbA1c (*p* ≤ 0.05) (Supplementary Figure 7).

Two studies reported that the addition of resistant starch 2 to the diet, had no significant effect on HOMA-IR (36, 38) or HOMA-%B and -%S (36). However, Ble-Castillo et al. (37) reported an increase in HOMA-IR after resistant starch supplementation. Peterson et al. (39), found a significant reduction in HbA1c after supplementation of 45 g resistant starch type 2.

## Discussion

### Resistant starch and acute glycemic response

Acute interventions were identified for resistant starch types 1–3. The present study suggests that both resistant starch type 1 (intact plant cell wall structures) and 2 (high-amylose starch) can significantly lower postprandial glucose in individuals with T2D or prediabetes, similar to what is observed in healthy subjects (28, 33). Interestingly, the subgroup analyses found that resistant starch type 2 significantly affected postprandial insulin. Resistant starch type 3 did not affect glycemia. Furthermore, resistant starch appears to affect postprandial glucose and insulin more potently in subjects with T2D, than in prediabetes. However, it is worth noting that these results are based on a relatively small pool of studies.

Resistant starch type 1 (intact plant cell structure) is found in starchy ingredients with intact kernels (41, 45, 46, 57, 63) or foods which have undergone less mechanical processing to maintain a large particle size (42, 47, 48, 56). Intact plant cell wall structures may inhibit enzymatic degradation and reduce the rate and extent of starch digestion and glucose absorption in the small intestine (9, 13). Additionally, resistant starch type 1 may increase intestinal viscosity and have higher concentrations of phenolic compounds, found within whole grains, which contribute to a lower rate of digestion (72, 73).

Resistant starch type 2 is often purified and added to baked products (43, 49, 51, 52, 54, 61) or supplemented (50). Whereas resistant starch type 3 is found in starchy foods that are cooked and cooled (53, 55). The dilution of total carbohydrate content with indigestible starch by adding resistant starch type 2 or 3 has an attenuating effect on postprandial glucose response (49, 51, 52, 54). However, few studies reported whether there were differences in the available carbohydrate content between the intervention and control (42, 49, 54). Furthermore, Giacco et al. (52) argued that the change in digestible starch (–16%) could not solely account for the magnitude of change in the glucose iAUC (–30%). Prior research has also suggested that resistant starch could induce a “second-meal effect” where the glycemic response to the subsequent meal is dampened (74). However, studies investigating this phenomenon did not find any significant impact on subsequent meal response (43, 52, 54).

There was substantial interstudy heterogeneity and a likelihood of publication bias in the acute postprandial meta-analyses. As heterogeneity decreased after subgroup analysis, heterogeneity could be partially attributed to differences in metabolic response between those with prediabetes and T2D and types of resistant starch.

### Resistant starch and chronic glycemia

Chronic interventions were identified for resistant starch types 1, 2, and 4. Periods of supplementation for these interventions spanned 1 day to 1 year. Resistant starch types 1 and 2 significantly lowered postprandial glucose, similar to results seen in acute studies. Resistant starch type 1 lowered postprandial insulin concentrations, whereas resistant starch type 2 did not. Interestingly, postprandial insulin trended toward an increase in resistant starch type 1 interventions whereas resistant starch type 2 appears to have the opposite effect. Resistant starch type 4 did not affect postprandial glycemia. Chronic interventions using resistant starch type 2 lowered fasting glucose and insulin but no effect was seen after chronic consumption of resistant starch type 1 and 4. The lack of significant effect seen could be due to the insufficient duration of resistant starch type 1 interventions and the small sample size for resistant starch type 4 interventions. Notably, the resistant starch type 1 study with the longest intervention (56 days), reported that intact grain structures improved pancreatic β-cell function and glucose-stimulated insulin secretion whilst refined grain structures did not (57). Subgroup analyses indicated that chronic resistant starch interventions improve fasting glucose and insulin in subjects with T2D, but not prediabetes. Dosage of resistant starch 2 and 4, ranged from 6 to 40 g per day. However, resistant starch type 1 content was not reported, making it difficult to determine whether these studies could be compared to resistant starch type 2–4 interventions.

Changes in postprandial glucose response seen for chronic interventions may be due to lowered available carbohydrate content and inhibited starch digestion, as the results were similar to that seen for the acute studies. Increased resistant starch delivery to the distal gut would elevate SCFA yield from gut bacteria fermentation, which may contribute to chronic improvements in postprandial and fasting glycemia (75, 76). SCFAs can bind to free fatty acid receptors 2 and 3 to promote the secretion of GLP-1, an incretin, from colonic L-cells (77, 78). GLP-1 promotes insulin secretion and glucose disposal in peripheral tissues, facilitating glucose homeostasis (79). Elevations in circulating acetate (50), acetate and butyrate (61) or propionate, butyrate and GLP-1 (58) concentrations were reported after resistant starch supplementation. Further research in healthy volunteers reports increases in GLP-1 secretion after a resistant starch intervention (80). In one intervention, researchers identified no significant difference in SCFA concentrations, possibly due to the short study duration (4 days) (38). Furthermore, no significant changes to glycemia were reported after 4 weeks but in a similar 12 weeks study, where the same dose (40 g/day) of resistant starch was given, significant improvements were seen (58, 59). Given this, one may assume the length of intervention could determine the extent of the metabolic adaptations seen.

Changes in glucose metabolism and insulin sensitivity can be confounded by weight loss or changes in body composition (81). Although many studies reported no significant change (58, 59, 64, 65, 67, 68), significant weight loss was recorded in some (37, 66, 69), one of which also reported the greatest reduction in fasting glucose (69).

### Comparison of metabolic outcomes between resistant starch types

Our results and a limited pool of published literature indicate that different types of resistant starch may differentially exert their effects on metabolic response *via* distinct mechanisms (16, 22, 82). For example, research has shown resistant starch type 3 may treat T2D by regulating the TCA cycle, amino acid and lipid metabolism (82). Additionally, few studies have suggested that certain resistant starches, e.g., resistant starch type 4, may have more profound effects on glycemia than others (22, 83). However, there is a dearth of research comparing multiple resistant starch types and glycemic responses. From the present study, resistant starch type 2 appears to have a more potent effect on postprandial response and fasting glucose concentrations. Resistant starch type 2 is easily added to food and can be consumed in larger quantities. Therefore, dose size could partially explain why resistant starch type 2 appears to have a more potent effect on glycemia. However, resistant starch type 1 content cannot be confirmed as it was not reported in these interventions. Indeed, the availability of resistant starch type 1 is dependent on its structural integrity which can be damaged during processing and digestion. Prior studies using finely or coarsely milled flours have found similar amounts of resistant starch preserved within the ileal fluid of both interventions (7, 34). However, differences in glycemic response are still seen, likely due to the presence of intact cells which can inhibit α-amylase activity and protect starch from digestion. One of the challenges is that little is known of the impact of cell wall integrity and how it affects the resistant starch content as the food is digested (84). Furthermore, resistant starch type 1 content varies among plant sources. For instance, structure-function studies have shown that legumes and wheat respond differently to processing and have distinct digestion profiles (12). Thus, distinct processing strategies could be warranted for targeting different plant sources to increase or preserve resistant starch type 1 content.

Additional RCTs using Omics, are required for a broader understanding of starch digestion in the gastrointestinal tract and the mechanisms regulated by resistant starch. However, the simple method of selecting high-resistant starch foods to reduce digestible carbohydrate content could improve glucose homeostasis in individuals with T2D, slowing disease progression and lowering the risk of comorbidities, like cardiovascular disease (85–87).

### Limitations and future recommendations

This review and meta-analysis has its limitations. Few studies explored GLP-1, GIP and insulin sensitivity which contributed to the lack of significance in the meta-analysis, underlining the need to explore the role of incretins in interventions investigating resistant starch and glycemic response. Although this review demonstrates that resistant starch significantly lowers glycemia, it is debatable whether these results would incur clinically significant improvements in glucose or insulin concentrations. It appears that postprandial glucose is attenuated when resistant starch replaces rapidly digestible starch, however, most RCTs did not report digestible starch content. Furthermore, resistant starch type 1 studies did not quantify resistant starch, making it difficult to determine whether it was given in similar quantities to resistant starch types 2–4. The authors also mention that differences in metabolic effects of resistant starch type 1 may be dependent on whether it is derived from grains or legumes. Unfortunately, interventions using legumes as a source of resistant starch type 1 were not included in this systematic review as there were no studies which fit the PICOS criteria. Additionally, adherence to the study was not reported in most chronic interventions, which may increase the risk of bias (Domain 2.1). Lastly, side effects can arise from substantial increases in resistant starch intake. However, as only one study reported side effects, this risk could not be assessed. Gastrointestinal tolerance must be recorded to determine the feasibility of resistant starch as a treatment for improving glycemic response. Larger studies with longer durations which explore the different resistant starch types are recommended.

Conclusions from this systematic review and meta-analysis should be drawn with caution. Significant heterogeneity was detected in several meta-analyses. Furthermore, a number of the included studies had a high risk of bias and sensitivity analyses show that once these studies were omitted, one outcome (acute resistant starch type 1 and postprandial insulin), was no longer significant. Furthermore, publication bias was detected in acute postprandial glucose, GIP and HbA1c meta-analyses.

## Conclusion

In this meta-analysis, the effect of resistant starch within intact plant cell structures and modified starch molecules on glycemia was comparable. There appears to be an argument in favor of dietary interventions using resistant starch types 1 or 2 for individuals with T2D, however, results were less conclusive in those with prediabetes. Chronic resistant starch type 1 and 2 interventions lowered postprandial glucose whereas solely chronic resistant starch type 2 interventions influenced fasting glucose and insulin. The difference in response between resistant starch types could reinforce the notion that resistant starch types exert their effects on glucose homeostasis *via* different mechanisms.

## Author contributions

JP and MC wrote the introduction and conducted the meta-analysis. JP designed the research. JP, MC, and NA screened the articles and conducted the data extraction. All reviewers and GF interpreted the findings and wrote the discussion. All authors read and approved the final manuscript.

## Footnotes

1
https://automeris.io/WebPlotDigitizer/
