# Supplementary material for: A comparison of the effects of resistant starch types on glycemic response in individuals with type 2 diabetes or prediabetes: A systematic review and meta-analysis
Source: Front Nutr. 2023 Mar 27;10:1118229. doi: 10.3389/fnut.2023.1118229 (PMC10085630; doi:10.3389/fnut.2023.1118229)
Supplement: Supplementary file 1 [file Data_Sheet_1.docx]

***Supplementary material***

**Supplemental figure 1**

**
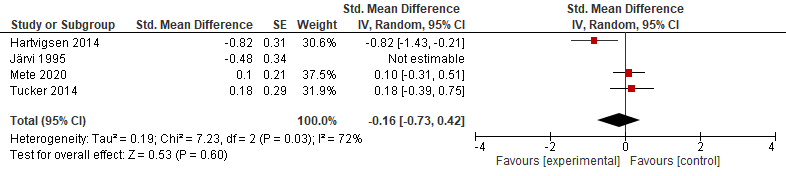
**

**Supplemental figure 1.** Sensitivity analysis of acute resistant starch type 1 and postprandial insulin AUC

**Supplemental figure 2**


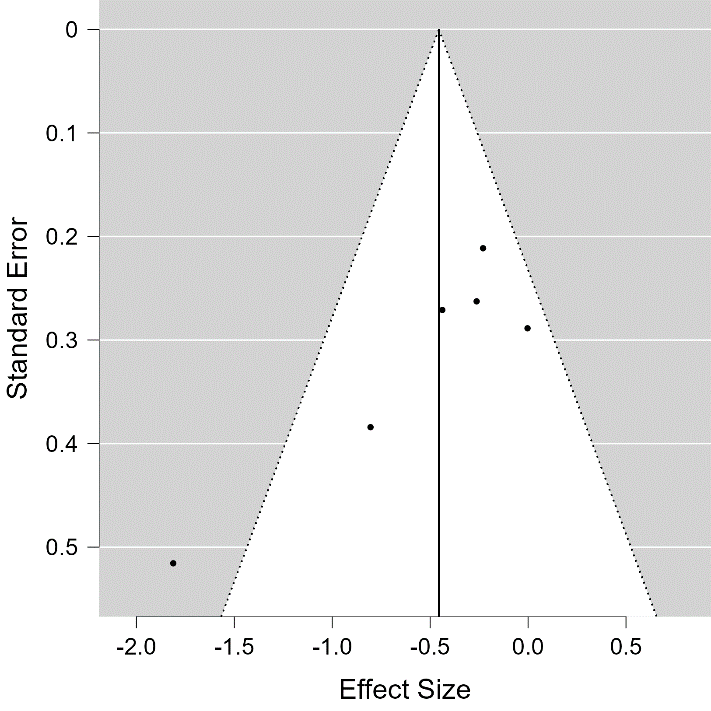


**Supplemental figure 2.** Funnel Plot of resistant starch type 1 and glucose postprandial AUC. Regression tests for functional asymmetry “Egger’s test” (P=0.005)

**Supplemental figure 3**


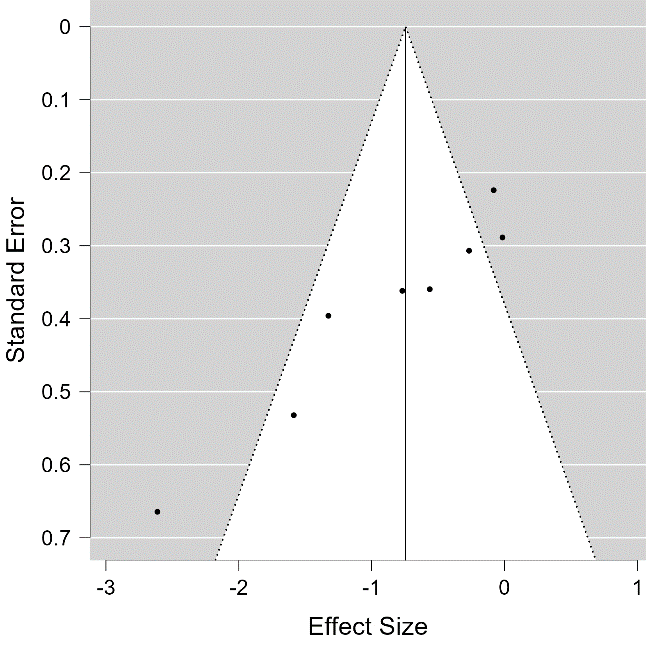


**Supplemental figure 3.** Funnel Plot of acute resistant starch type 2-4 and postprandial glucose AUC. Regression tests for functional asymmetry “Egger’s test” (P=<0.001).

**Supplemental figure 4A**

**
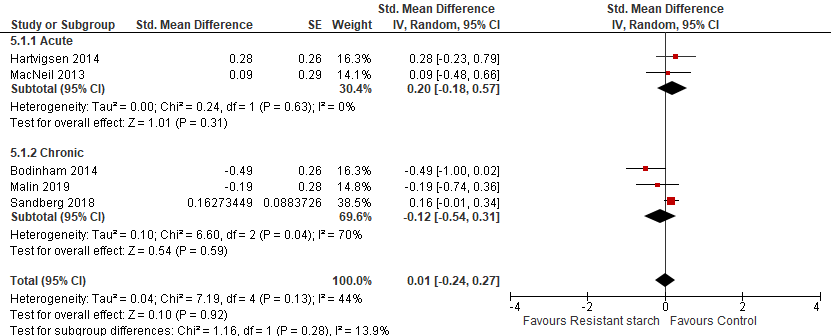
**

**Supplemental figure 4B**

**
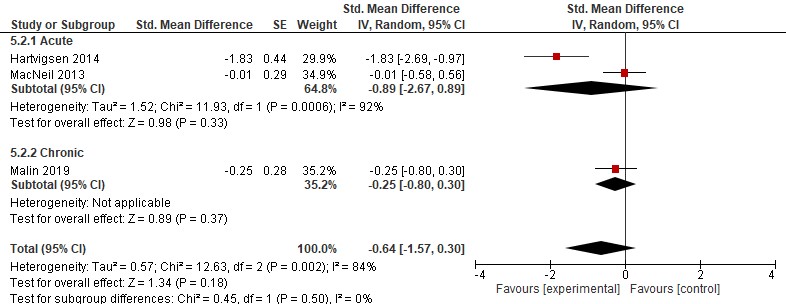
**

**Supplemental figure 4.** Meta-analysis results for resistant starch intake on postprandial GLP-1 (A) and GIP (B) in patients with T2D and prediabetes, with subgroup analysis for acute and chronic interventions.

**Supplemental figure 5**


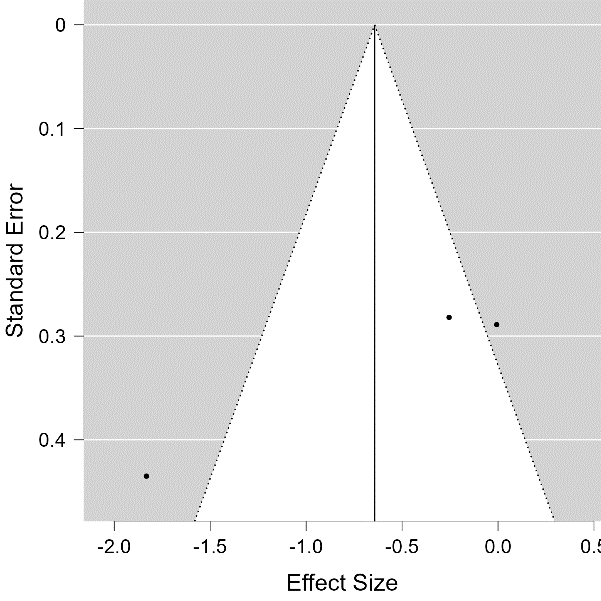


**Supplemental figure 5.** Funnel Plot of GIP. Regression tests for functional asymmetry “Egger’s test” (P=<0.001)

**Supplemental figure 6A**

**
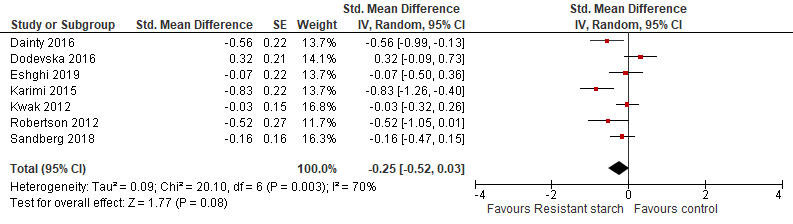
**

**Supplemental figure 6B
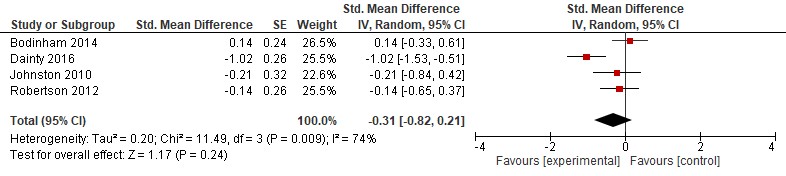
**

**Supplemental figure 6C
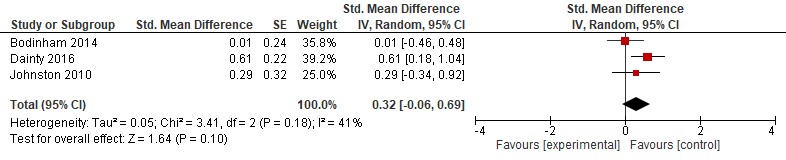
**

**Supplemental figure 6D**

**
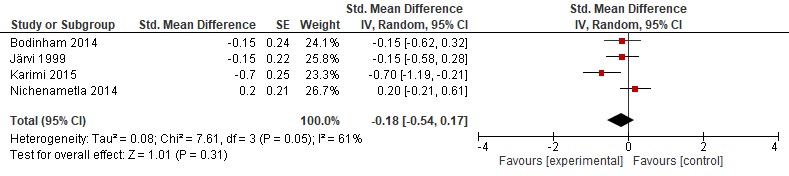
**

**Supplemental figure 6.** Meta-analysis results for resistant starch intake on HOMA-IR (A), HOMA-%B (B), on HOMA-%S (C), and HbA1c (%) (D) in patients with T2D and prediabetes.

**Supplemental figure 7**


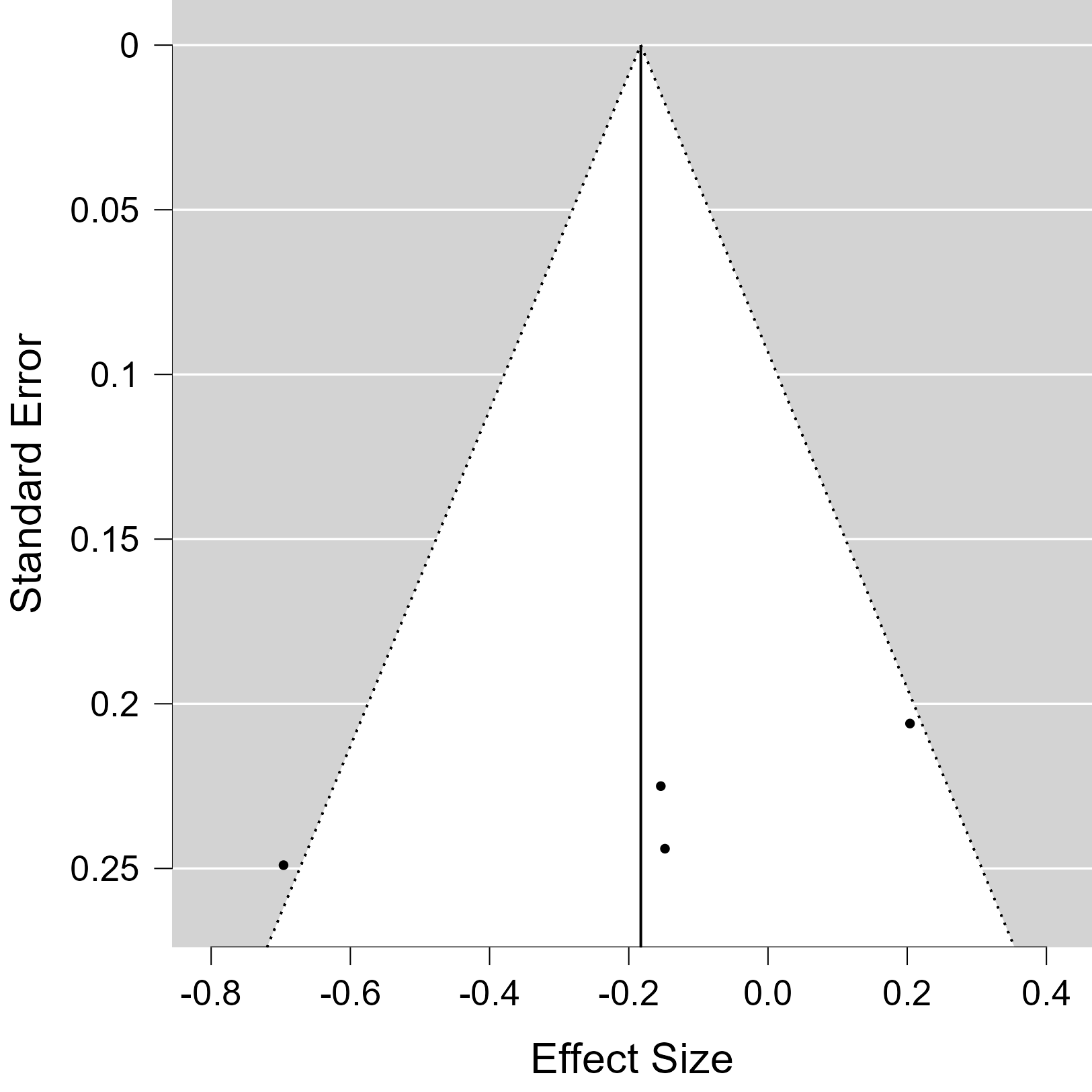


**Supplemental figure 5.** Funnel Plot of HbA1c. Regression tests for functional asymmetry “Egger’s test” (P=0.0015)
